# Antioxidant Response in Skeletal Muscle

**DOI:** 10.3390/antiox14121445

**Published:** 2025-11-30

**Authors:** Elżbieta Supruniuk, Jan Górski

**Affiliations:** 1 Department of Physiology, Medical University of Białystok, 15-222 Białystok, Poland; 2 Department of Medical Sciences, Academy of Applied Sciences, 18-400 Łomza, Poland; jan.gorski@umb.edu.pl

## 1. Introduction

Skeletal muscle, which in men accounts for nearly half of the body’s mass, stands out as the most adaptable and energetically demanding tissue. In addition to facilitating mobility and maintaining posture, it is indispensable for regulating the body’s overall metabolism and communicating with distant organs by releasing myokines. To perform these functions, muscles continuously adjust their structure and biochemical activity, which requires a precisely tuned equilibrium between energy generation, repair mechanisms, and cellular turnover. This remodeling of skeletal muscle is governed by its redox environment, which is determined by the balance between free radical production and the antioxidant systems that regulate it [1].

To control skeletal muscle function and metabolic flexibility, a well-balanced redox network is established, in which endogenous antioxidant systems and exogenous inputs interact across molecular, cellular, systemic, and temporal levels. At moderate concentrations, reactive oxygen and nitrogen species (RONS) provide the modulatory stimulus for cell adaptation, promoting exercise tolerance, mitochondrial remodeling, and stress resistance. A central hub in maintaining this equilibrium is nuclear factor erythroid 2-related factor 2 (Nrf2), which orchestrates the expression of antioxidant and detoxifying enzymes [2]. Oxidative stress occurs when RONS accumulate more rapidly than they can be neutralized. Although these molecules are intrinsic byproducts of normal metabolism, excessive levels can damage cellular constituents, including lipids, proteins, and DNA/RNA. Heightened RONS production and weakened antioxidant defenses are common features of muscle wasting, disuse, and age-related deterioration. Fast-twitch fibers, due to their high metabolic demands and relatively limited protective systems, show particular sensitivity to oxidative challenges [3].

This Special Issue focused on mechanisms by which redox processes accomplished by both endogenous antioxidant systems and exogenous antioxidant inputs govern muscle function. Because skeletal muscle repeatedly endures mechanical load and metabolic demand, redox dynamics are further challenged by factors such as intense or prolonged exercise, periods of inactivity, aging, nutritional variation, and systemic stress. Such influences modify the oxidative environment of muscle and ultimately determine whether redox signals support beneficial adaptive changes. It is essential to investigate the mechanisms by which intrinsic antioxidant defenses interact with applied stressors to maintain redox equilibrium.

## 2. Overview of Published Articles

### 2.1. Acquired Endogenous Defense Mechanisms: The Muscle’s Intrinsic Redox Machinery

Skeletal muscle has developed sophisticated defense mechanisms, involving redox-mediated pathways, that allow it to adapt and even thrive under various insults, including metabolic and mechanical stressors, to protect cells against oxidative stress and resulting inflammation. Pribil Pardun et al. [Contribution 1] showed that contractile activity evoked by electrical stimulation (EPS) activates the Nrf2-dependent antioxidant pathway in C2C12 myotubes. This leads to the activation of thioredoxin and glutathione systems to the greatest extent that persisted for up to 24 h after EPS, and in turn protected the cells against hydrogen peroxide-induced cytotoxicity. Rather than being purely deleterious, moderate ROS production served as a signal that reinforced redox buffering capacity, improving mitochondrial efficiency and resilience, and controlling the degree of inflammation. This illustrates the concept of exercise-induced hormesis, wherein a transient increase in low levels of a stressor (e.g., radicals) exerts a beneficial effect on the cells [4]. However, the implementation of blood flow restriction (BFR) training protocols has added an important cue in adjusting skeletal muscle response to oxidative stress in training. Direct measurements of free radical production confirmed a systemic increase in ROS after BFR exercise associated with local reactive hyperemia, which contributed to increased muscle mass, strength, and performance [5].

The dual role of RONS in exercise was reviewed by Meng et al. [Contribution 2] based on the data from elite soccer players. They emphasized the need to monitor individuals’ oxidative balance, training load, as well as physiological and psychological indicators of perceived exertion to personalize effective recovery and nutrition strategies. Antioxidant supplementation has been commonly used to overcome oxidative stress-related muscle soreness and damage. However, the timing and dosing of exogenous antioxidants require careful management to prevent blunting the adaptations that depend on repetitive exposure to redox-signaling activation, which supports force production, improves metabolic flexibility, delays fatigue development, and enhances muscle regenerative capacity. Variability in responses can be caused by genetic polymorphisms in antioxidant genes (such as SOD2, GPX1, or CAT), seasonal fluctuations in buffering capacity, dietary habits, and training history.

Sutton et al. [Contribution 3] highlighted the crosstalk between circadian rhythms and redox homeostasis, especially in the case of muscle aging. Circadian clock, governed by the transcriptional feedback loop of brain and muscle ARNT-like protein 1 (BMAL1) and circadian locomotor output cycles kaput (CLOCK), coordinates daily oscillations in metabolism, mitochondrial function, and antioxidant defenses. For instance, approximately 38% of mitochondrial proteins exhibit diurnal oscillations, while some of them were abolished by a high-fat diet [6]. With advancing age, circadian amplitude diminishes, and the rhythmic expression of redox genes becomes blunted. The authors stated that this desynchronization contributes to the deterioration of skeletal muscle function with age by impairing mitochondrial turnover and reducing resistance to oxidative stress. Moreover, they propose that interventions targeting circadian alignment, involving time-restricted feeding, light exposure, and appropriately timed exercise, may restore redox synchrony and improve muscle health [Contribution 3].

### 2.2. Environmental and Physiological Stressors: Redox Imbalance as a Common Factor

Spaceflight represents a unique physiological model of extreme disuse and environmental stress, characterized by muscle unloading, fluid shifts, radiation exposure, and altered circadian cues. Blottner et al. [Contribution 4] analyzed skeletal muscle biopsies obtained from astronauts who had been on the International Space Station (ISS), comparing short-duration missions (~9 days) and long-term missions (>180 days). Proteomic analyses showed extensive S-nitrosylation of metabolic, mitochondrial, and contractile proteins, including creatine kinase, glycolytic enzymes, and the tricarboxylic acid (TCA) cycle components. These molecular alterations contribute to muscle atrophy independent of mechanical unloading. Interestingly, while short-duration missions induced a pattern of over-nitrosylation associated with reduced mitochondrial activity and impaired ATP production, long-duration missions showed a partial adaptation with a distinct nitrosoproteomic profile, suggesting that human muscle can adjust redox balance under sustained microgravity conditions. This adaptation, however, appeared incomplete, pointing to the limits of physiological plasticity in the absence of gravitational loading. What is more, following about 180 days of space flight, both the soleus and gastrocnemius muscles exhibited reduced fiber size and force, with the greatest atrophy occurring in the order: soleus type I > soleus type II > gastrocnemius type I > gastrocnemius type II [7]. The above also underlines parallels between muscle disuse due to microgravity or inactivity (bed rest, aging, immobilization). It was demonstrated that antioxidant administration protects from muscle fibrosis via attenuating immobilization-induced activation of profibrogenic transforming growth factor β1 (TGF-β1) signaling [8]. Nevertheless, the direct mechanisms underlying ROS’s role in the development of muscle atrophy in disuse settings in humans remain putative.

Among environmental stressors, mycotoxins are widespread contaminants to which humans and animals are exposed via food, inhalation, or contact. Certain mycotoxins can be harmful by interfering with protein synthesis, preventing particle clearance of the lungs, damaging macrophage systems, raising sensitivity to bacterial endotoxins, and also inducing oxidative stress. Due to their heat-stability, complete eradication of mycotoxins is difficult despite the variety of procedures used, including physical separation, heat treatment, cleaning, milling, washing, radiation, biological or chemical agents, and extraction with solvents [9]. One of the most common mycotoxins is zearalenone, which is present in a range of food products, including cereals, dried fruits, and spices [10]. Li et al. [Contribution 5] presented a mechanistic analysis of skeletal muscle injury in response to zearalenone administration to mice. The authors observed that this mycotoxin damages skeletal muscle through excessive ROS generation, mitochondrial dysfunction, and apoptosis. This resulted in reduced myoblast proliferation, differentiation, and slow-to-fast myofiber shift. In the long run, such muscle phenotypic changes could lead to reduced endurance and fatigue resistance, impaired muscle performance, and increased muscle atrophy [11]. Importantly, co-treatment with antioxidants such as glutathione, nicotinamide mononucleotide, or melatonin mitigated these pathological manifestations, restoring redox equilibrium and muscle integrity [Contribution 5].

### 2.3. Restoring Redox Balance Through Bioactive Compounds

Herbal medicine offers potential therapeutic strategies to prevent or reverse muscle atrophy, as explored by Kim et al. [Contribution 6]. The ethanol extract of Jakyak-Gamcho-Tang exerts potent muscle-protective effects by inhibiting C2C12 myotubes degradation when exposed to H_2_O_2_, dexamethasone, or palmitic acid. The superior beneficial effects of ethanol extract compared to predominantly used water-based extraction were attributed to the higher concentration of key bioactive compounds. The authors identified paeoniflorin, isoliquiritigenin, catechin, glycyrrhizic acid, and glabridin as main substances regulating mitochondrial function, ROS release, and muscle-atrophy-related signaling cascades. Transcriptomic analysis upregulation of genes associated with oxidative phosphorylation, mitochondrial biogenesis, peroxisome proliferator-activated receptor γ coactivator 1α (PGC-1α) signaling, mammalian target of rapamycin complex 1 (mTORC1) signaling, and estrogen-related receptor α (ERRα) targets, while downregulation in muscle-atrophic TGF-β signaling. The study underscores the need for standardization and optimization of herbal medicine to enhance therapeutic efficacy.

In line with those findings, Iannuzzo et al. [Contribution 7] tested the biological activity of *Rhodiola rosea* extracts obtained from wild to controlled cultivation in primary human myocytes. The authors showed that optimization of cultivation conditions resulted in a unique phytochemical profile that strengthened the antioxidant properties, being an essential step for integrating herbal agents into evidence-based antioxidant therapy. *Rhodiola rosea* preserves cellular homeostasis when challenged by oxidative load, possibly underpinning its beneficial effects on muscle performance and mitigating fatigue. The plant’s role in growth and repair of muscle was also associated with the stimulation of ATP production and improved mitochondrial respiration. Moreover, the interconnection between antioxidant mechanisms and the upregulation of folate and polyamine pathways in controlled cultures may contribute to improved muscle regeneration after injury or recovery from physical exercise. By regulating redox-sensitive pathways and maintaining cellular homeostasis, *Rhodiola rosea* emerges as a promising nutraceutical for supporting muscle health and combating oxidative stress-related dysfunction [Contribution 7]. These biological properties can be especially useful in sarcopenia management, a disease characterized by accelerated loss of skeletal muscle mass and strength, usually affecting the aging population [12]. Another study by Wojszel et al. [Contribution 8] explored the relationships between redox balance and two muscle-derived myokines implicated in neuro-muscular crosstalk, such as irisin and brain-derived neurotrophic factor (BDNF), in patients over 60 years of age. These molecules participate in exercise-related adaptations by supporting substrate delivery to the contracting muscle. The authors showed that reduced circulating irisin and BDNF levels correlate with impaired antioxidant defenses and elevated oxidative stress markers, which may underlie neuromuscular decline in the elderly. Dynapenia is therefore related to a disruption in systemic communication between muscle and brain metabolism, which represents a significant clinical challenge.

## 3. Conclusions

The studies collected in this Special Issue highlight the central role of redox processes in maintaining skeletal muscle function and adaptability across the lifespan. Transient increases in RONS during contractions act as signals to enhance antioxidant defenses, stimulate mitochondrial remodeling, and promote adaptation. On the other hand, chronic or unmitigated oxidative and nitrosative stress, as seen during aging, unloading, or metabolic dysfunction, overwhelms defenses and drives atrophy. Notably, targeted strategies such as antioxidant supplementation could abolish training benefits resulting from intrinsic adaptive processes. For instance, redox responses to sprint interval training were blunted in type 2 fibers due to antioxidant provision, underscoring that interval training is effective without supplementation and challenging the common belief that antioxidants are universally beneficial in exercise contexts [13]. Redox dynamics are influenced by circadian alignment, training load, nutrition, genetic background, and environmental exposures, indicating that timing and personalization are crucial for effective interventions. Advances in computational modeling and omics integration provide tools to map key nodes in antioxidant networks, offering the possibility of predicting muscle responses and adjusting strategies to individual redox signatures.

## Abbreviations

The following abbreviations are used in this manuscript:

BDNF	brain-derived neurotrophic factor
BFR	blood flow restriction
CAT	catalase
ERRα	estrogen-related receptor α
GPX1	glutathione peroxidase 1
mTORC1	mammalian target of rapamycin complex 1
Nrf2	nuclear factor erythroid 2-related factor 2
PGC-1α	peroxisome proliferator-activated receptor γ coactivator 1α
SOD2	superoxide dismutase 2, mitochondrial
TGF-β	transforming growth factor β
